# The Role of LGBTQ+ Vicarious Trauma in Eating Disorder Risk—A Psychological Parallel Mediation Model

**DOI:** 10.3390/bs15101343

**Published:** 2025-09-30

**Authors:** Fabrizio Santoniccolo, Tommaso Trombetta, Maria Noemi Paradiso, Luca Rollè

**Affiliations:** 1 Department of Psychology, University of Turin, 10124 Turin, Italy; fabrizio.santoniccolo@unito.it (F.S.); marianoemi.paradiso@unito.it (M.N.P.); l.rolle@unito.it (L.R.); 2 School of Health, Medicine and Life Sciences, University of Hertfordshire, Hatfield AL10 9AB, UK; 3 Institute of Applied Psychology, University of Social Science, 90-229 Łódź, Poland

**Keywords:** minority stress, eating disorders, disordered eating, body image, vicarious trauma, emotion regulation, self-esteem, shame, health disparities

## Abstract

Minority stress appears to be consistently associated with a heightened risk of developing eating disorders. There is limited data investigating the role of witnessed heterosexist experiences (vicarious trauma), such as discrimination, harassment, or violence happening to other LGBTQ+ people. The present study aims to examine the association between vicarious trauma and eating disorder risk while surveying the mediating role of emotional dysregulation, self-esteem, and shame. An anonymous online survey was conducted involving 376 LGBTQ+ people from Italy. Participants completed self-report questionnaires regarding heterosexist experiences and factors associated with eating behavior. Descriptive, bivariate, and mediation analyses were conducted using the “PROCESS” macro. Statistically significant positive associations were found between all the main variables in bivariate analyses. Mediation analyses highlighted a direct effect of vicarious trauma on eating disorder risk and indirect effects of vicarious trauma on eating disorder risk through low self-esteem and emotion dysregulation. The indirect effect through shame was nonsignificant. Vicarious trauma appears to have a significant direct effect on eating disorder risk and small but significant indirect effects through emotional dysregulation and low self-esteem. Health promotion contexts would benefit from policies at the institutional, organizational, and social levels to prevent minority stress and reduce observed health disparities.

## 1. Introduction

### 1.1. Prevalence and Impact of Eating Disorders

Eating disorders are a serious risk to people’s physical and mental health and have been found to be among the mental illnesses with the highest mortality risk, healthcare burden, and years lived with disability (Van Hoeken & Hoek, 2020), as well as a significant impairment of quality of life (Ágh et al., 2016). Eating disorders include a heterogeneous set of mental health conditions, such as anorexia nervosa, bulimia nervosa, binge eating disorder, and other feeding disorders, each of which appears to have a different etiopathology (Agras & Robinson, 2017), although some risk factors appear to be common to most (Jacobi et al., 2017). The global burden from these disorders has been increasing and has been forecasted to rise further (Liu et al., 2025). They feature significant comorbidities with other mental health conditions such as anxiety disorders, mood disorders, and substance abuse (Juli et al., 2023), posing a significant risk to people’s wellbeing. The Italian context has been estimated to have a relatively high prevalence of eating disorders among both adolescents (D’Anna et al., 2022) and adults (Carta et al., 2014).

### 1.2. Minority Stress and Health Disparities in LGBTQ+ People

Heterosexist experiences can be defined as negative social experiences that a person can have due to the fact they deviate from a cisgender and/or heterosexual identity—several forms can be identified (Balsam et al., 2013). An expanding body of research has highlighted that, when compared to cisgender and heterosexual people, LGBTQ+ people show a heightened risk of development of psychological symptoms as well as generally worse outcomes in mental health across different domains (Clark et al., 2024; Khanolkar et al., 2023; Trombetta & Rollè, 2023). Despite a recent increase in LGBTQ+ acceptance by the general population in Western societies (Wittgens et al., 2022), heterosexist experiences appear to be still prevalent. The health disparity between LGBTQ+ people and cisgender/heterosexual people has been hypothesized to be caused by minority stress—the presence of additional stress factors that are specific to LGBTQ+ people (Meyer, 2003; Meyer & Frost, 2012). These may include distal factors such as discrimination and violence as well as proximal factors such as internalized negativity towards one’s sexual identity. Over the decades since its publication, the minority stress model has received very good support for its hypotheses in a variety of mental health fields (Frost & Meyer, 2023) and has been expanded, among other contributions, through the use of a psychological mediation framework (Hatzenbuehler, 2009). The framework attempts to explain how exactly minority stress causes the health disparities found in scientific literature, particularly by surveying the impact of stigma, discrimination, and violence against general psychological processes (e.g., interpersonal, social, cognitive, and coping processes) and how this impact may, in turn, heighten psychological symptoms. This framework has been successfully used in different types of stigma, including weight stigma (Sikorski et al., 2015) and sexual-orientation-related internalized stigma (Trombetta et al., 2023; Trombetta & Rollè, 2024).

### 1.3. How Minority Stress Impacts Eating Behaviors

Consistently with minority stress theory, LGBTQ+ people have been found to show a health disparity in the risk of eating disorders when compared with cisgender/heterosexual people (Parker & Harriger, 2020). In particular, the risk has been estimated to be twice that of cisgender/heterosexual people, and preliminary evidence suggests that it may be significantly higher in people that have experienced discrimination (Kamody et al., 2020). This could theoretically also be compounded by multiple marginalizations (Burke et al., 2020; Crenshaw, 1991), as previously explored regarding socioeconomic status (Burke et al., 2023), age (Jahrami et al., 2019), and weight stigma (Himmelstein et al., 2017).

Several studies have found minority stress to play a significant role in determining a heightened risk of several disordered eating behaviors, impacting several intrapsychic characteristics in the process that may explain this relationship as mediators, such as body surveillance, body shame, and negative affect (Santoniccolo & Rollè, 2024). However, the mediating role of several intrapsychic aspects that are key risk factors in determining eating disorders remains unexplored. Factors such as emotion regulation, self-esteem, and shame have been highlighted as important in causing and maintaining disordered eating (Clausen et al., 2011) but have received little attention in minority stress literature. In particular, emotion regulation is a known across-the-board risk factor for general psychological symptoms (Gross et al., 2019) and eating disorders specifically (Leppanen et al., 2022). Feelings of shame (Nechita et al., 2021) and low self-esteem (Kästner et al., 2019) are also known associated factors with eating disorders, and both may be affected by minority stress due to possible negative self-evaluation stemming from heterosexist experiences.

### 1.4. LGBTQ+ People in the Italian Context

In the Italian context, public attitudes towards LGBTQ+ people have been slowly improving over the decades, despite showing some heterogeneity. EURISPES’ data shows a generally favorable trend of attitudes toward LGB rights. The trend for marriage in same-gender couples is favorable, growing from roughly 59.2% of respondents in favor in 2015 to 66.8% in 2025. Attitudes towards same-gender couple adoption are slightly less favorable but show a much sharper growth from 27.8% in 2015 to 51.9% in 2025. However, attitudes towards rights for trans and gender-diverse people are much less favorable and still far from showing general acceptance. Legal gender affirmation based on self-identification without any medical certification measures a favor of 37.2% in 2025. Similarly, recognition of non-binary gender identities shows a favor of 51.1% in 2025 (EURISPES, 2015, 2025). Additionally, in the 2025 IPSOS Pride Survey, attitudes regarding laws and policies promoting LGBTQ+ rights were overall favorable—64% of Italian respondents were in favor of laws banning discrimination against LGBTQ+ people in general, 78% regarding the protection from discrimination of trans people in particular. Furthermore, 61% of respondents were in favor of allowing trans people to use single-gender facilities (e.g., public restrooms), while 53% were in favor of including an option other than “male” and “female” for identity documents (IPSOS, 2025).

The Italian context has however consistently been lagging behind these relatively favorable attitudes when considering the enactment of legislation and policies.

In the Rainbow Map developed by ILGA Europe, Italy ranks 35th out of 41 European countries in 2025, with a global score of 24%, far below the European average of 41% (ILGA Europe, 2025). Several key LGBTQ+ rights, laws, and policies have been implemented partially or not at all (Equaldex, 2025). Homonegative experiences are additionally still very persistent (Callahan & Loscocco, 2023), and experiences of discrimination against LGBTQ+ people remain exceedingly common (ISTAT, 2023). Previous studies on minority stress in the Italian context confirm these data points, painting it as a prevalent experience with important consequences for mental health (Baiocco et al., 2023; Priola et al., 2014; Rosati et al., 2024; Scandurra et al., 2017, 2019, 2020).

### 1.5. The Experience of Vicarious Trauma

Vicarious trauma can be defined as a proximal stressor that a person experiences due to sharing identity aspects with people that are subjected to violence, discrimination, or unfair treatment. This concept differs from secondary traumatic stress, which refers to trauma stemming from experiencing graphic details of other people’s traumatic experiences (Sprang et al., 2019). The experience of vicarious trauma in this paper specifically refers to witnessing or hearing about heterosexist experiences happening to other LGBTQ+ people (Balsam et al., 2013). In this instance, vicarious trauma also implies a general concept of the experience of traumatization that does not refer to or imply a specific diagnostic category (e.g., post-traumatic stress disorder). Hearing of heterosexist experiences that have happened to other people may contribute to the feeling of existing in a social climate that is negative towards this part of one’s sexual identity as well as cultivate in LGBTQ+ people the feeling that the mere fact of being openly LGBTQ+ is socially stigmatized, makes one vulnerable to mistreatment, and can even be a lethal threat to one’s safety. This can result in a state of hypervigilance in anticipation of possible environmental threats, which can adversely affect people’s wellbeing (Rostosky et al., 2022). A representative example is hearing about hate crimes happening to other LGBTQ+ people (Noelle, 2002), which can have rippling effects throughout the victim’s social circles and end up negatively affecting those who share this part of their identity as well (Iganski, 2001). Vicarious trauma can therefore be conceptualized as a heterosexist experience in its own right.

Despite being an unfortunately frequent part of minoritized populations’ lives, this phenomenon has received relatively little attention, remaining an understudied area.

### 1.6. Aims

To the authors’ knowledge, no studies have been conducted that survey the role of vicarious trauma in eating disorder risk or the role of possible mediating factors in this relationship. No studies of this kind have additionally been conducted in the Italian context.

Informed by the psychological mediation framework (Hatzenbuehler, 2009), this study aims to examine the relationship between vicarious trauma and eating disorder risk, exploring the possible mediating role of low self-esteem, emotion dysregulation, and general shame.

### 1.7. Hypotheses

**H_1_.**  *Vicarious trauma is positively and directly associated with eating disorder risk.*

**H_2_.**  *Vicarious trauma is positively and directly associated with feelings of shame, low self-esteem, and emotion dysregulation.*

**H_3_.**  *Feelings of shame are positively and directly associated with eating disorder risk.*

**H_4_.**  *Low self-esteem is positively and directly associated with eating disorder risk.*

**H_5_.**  *Emotion dysregulation is positively and directly associated with eating disorder risk.*

**H_6_.**  *Feelings of shame, low self-esteem, and emotion dysregulation mediate the relationship between vicarious trauma and eating disorder risk.*

**Figure 1:**
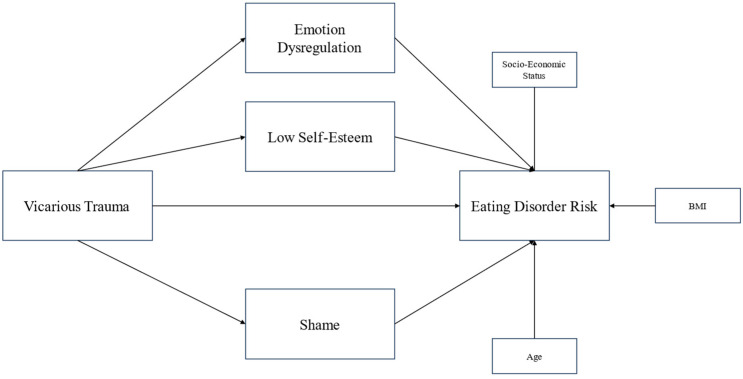
Hypothesized parallel mediation model. BMI: body mass index.

The hypothesized parallel mediation model is synthesized in Figure 1 (including covariates). Relationships between covariates and mediators have been omitted for simplicity.

## 2. Materials and Methods

### 2.1. Participants

The initial sample was composed of 386 participants in the “Factors associated with the eating behaviors of LGBTQ+ people” project carried out in Italy between June 2024 and April 2025.

Three inclusion criteria were defined:

- Identifying as any LGBTQ+ identity regarding sexual orientation or gender identity (e.g., gay, lesbian, bisexual, pansexual, queer, trans/transgender, non-binary, gender non-conforming, genderfluid, etc.);
- Being at least 18 years old;
- Being able to read, understand, and write in the Italian language.

One exclusion criterion was defined:

- Identifying as heterosexual while declaring being cisgender (i.e., having a gender identity that matches one’s assigned gender at birth).

### 2.2. Procedure

The study procedures were carried out in accordance with the ethical standards of the American Psychological Association and the 1964 Declaration of Helsinki. The study was approved by the Bioethical Committee of the University of Turin on 30 April 2024 with protocol number 0307736. An online survey was developed using the LimeSurvey platform running version 6.3.8+231204. The link to the survey was disseminated through public flyers at relevant places and events (e.g., LGBTQ+-friendly establishments, local Pride events), the researchers’ personal and professional networks, emails, and through collaboration with gyms, public libraries, and LGBTQ+ associations. The study also employed snowball sampling by asking the participants to send the link and/or flyer to other LGBTQ+ people they may know, disseminating the study through word of mouth. Participants were informed of the general topic of the study, along with possible risks and benefits connected to participation, asked to confirm being at least 18 years old, and asked for consent to participation. Participation was voluntary and completely anonymous. No reward or incentive was offered for participation in the study.

Participants were asked to fill out a sociodemographic sheet, in which they were asked which word best described their sexual orientation and gender identity, as well as to confirm whether they were a trans or otherwise gender-diverse person. Participants who reported both a heterosexual and cisgender identity were not allowed to participate in the study.

Participants responded to a battery of questionnaires surveying aspects related to experiences as LGBTQ+ people, body image, and eating behaviors. Whenever possible, validated questionnaires in the Italian language were used. The full questionnaire took participants an average of 30 min to complete.

A number of response quality control measures were employed. Two attention checks were added at random points throughout the survey. Participants who failed one or more attention checks were excluded from the analyses. The participants’ response time was also screened: responses that had significantly quicker response time compared to the mean were checked for response sets and incoherent responses. Participants also developed an anonymous participant code, which was used to screen for duplicate answers. Finally, participants who entered invalid or insufficient information in the “sexual orientation” or “gender identity” (e.g., using the “Other” field to enter an answer that is not relevant to the respective text field) were excluded. After this data quality screening, 376 participants were retained in the current analyses. Notably, the sample mostly comprised individuals with high education and with good socioeconomic status, which should be kept in mind as it may limit generalizability of the findings.

### 2.3. Measures

Eating Disorder Inventory—3 (EDI-3; Garner, 2004; Giannini et al., 2008): Participants responded to 91 items evaluating eating behaviors, attitudes, body image, and other psychological aspects connected to eating disorders. The EDI-3 uses a 5-point Likert scale assessing the participants’ perceived frequency of the relevant items from “never” to “always”. In this study, three subscales were used.

The low self-esteem subscale involves items related to a low evaluation of one’s subjective personal worth (such as «I feel inadequate» or «I feel secure about myself», reverse scored). Higher scores suggest lower self-esteem.

The emotion dysregulation subscale involves items related to one’s ability in recognizing and regulating one’s emotions (such as «Other people would say that I am emotionally unstable» or «I don’t know what’s going on inside me»). Higher scores suggest lower abilities in emotion regulation.

The Eating Disorder Risk Composite (EDRC) scale score is the sum of the scores from three additional subscales from the EDI-3: the Bulimia subscale, which measures behaviors and attitudes related to overeating (such as «I stuff myself with food»); the “Drive for Thinness” subscale, which measures one’s desire to attain a thinner body (such as «I am preoccupied with the desire to be thinner»); the Body Dissatisfaction subscale, which measures one’s current lack of satisfaction with body parts and their body as a whole (such as «I feel satisfied with the shape of my body», reverse scored). This composite scale is conceptualized as a general evaluation of eating disorder risk rather than pointing to a specific eating disorder. Higher total scores suggest higher general eating disorder risk.

Daily Heterosexist Experiences Questionnaire (Balsam et al., 2013): Participants responded to 38 items related to daily heterosexist experiences relevant to being LGBTQ+ specifically (some scales from the original were omitted). The scale allows participants to report on whether they had a specific experience or not. The scale was scored in the “Distress” mode, as participants reported on their perceived distress level for each experience (depending on the level of distress, ranging from “did not happen/does not apply to me” and “not at all”—both scored 1 point—to “extreme”—5 points). The scale was translated into Italian. For this study, the vicarious trauma subscale was used, which involves participants reporting about witnessing heterosexist experiences happening to other people (such as «Hearing about hate crimes (e.g., vandalism, physical or sexual assault) that happened to LGBT people you don’t know» or «Hearing other people being called names such as “fag” or “dyke”»). Higher scores correspond to more distressful heterosexist experiences related to vicarious trauma.

Personal Feelings Questionnaire—2 (PFQ-2; Di Sarno et al., 2022; D. W. Harder et al., 1993): The Italian version of the PFQ-2 was administered. The PFQ-2 has been used with LGBTQ+ people before (Scheer & Antebi-Gruszka, 2019) and has been found to have good psychometric properties (D. W. Harder et al., 1993; D. Harder & Zalma, 1990). The PFQ-2 comprises 22 items measuring a person’s tendency to experience feelings of guilt and shame by asking them to rate how frequently they experience each sensation (e.g., «Feeling humiliated» or «Feeling you deserve criticism for what you did») on a 5-point Likert-type scale ranging from “Never” to “All the time or almost always”. Raw scores were computed according to instructions for the Shame subscale. Higher scores are representative of more common feelings of shame.

Body Mass Index (BMI): using participants’ self-reported height and weight, their BMI (ratio between one’s mass and squared height) was computed.

### 2.4. Data Analysis

All data analyses were performed in RStudio using R version 4.5.1 (R Core Team, 2024). Two-tailed bivariate correlations between the variables were tested using Pearson’s correlation (r), and results were interpreted according to original guidelines. The PROCESS macro version 5.0 for R (Hayes, 2022) was used for mediation analysis, employing Model 4. The vicarious trauma score was entered as the independent variable. The low self-esteem, emotion dysregulation, and shame scores were entered as parallel mediators. The EDRC score was entered as a dependent variable. Age, BMI, and socioeconomic status were entered as covariates, in accordance with the literature highlighting their influence on sexual identity, body image, and eating behaviors.

Data was checked for consistency with assumptions of linear regression through visual checking of P-P and Q-Q plots and through checking of the Variance Inflation Factor (VIF). Due to the violation of the multi-normality assumption, a heteroskedasticity-consistent estimator was selected in PROCESS (HC3; Davidson & MacKinnon, 1993; Hayes & Cai, 2007). Bootstrap estimation with 5000 samples was used to determine 95% Confidence Intervals (CIs); effects were considered significant when CIs did not include 0.

#### Subscale Reliability

To estimate the reliability of the employed subscales, McDonald’s ω (McDonald, 2013) was computed using the OMEGA macro (Hayes & Coutts, 2020) through forced single-factor maximum likelihood analysis. Computed values are available in Table 1.

All employed subscales showed good or excellent reliability.

### 2.5. Missing Values

One value was missing regarding age, one value was missing regarding education status, and five values were missing regarding socioeconomic status. Due to the very small quantity of missing values, these were handled by replacing them with the sample’s median values.

## 3. Results

### 3.1. Descriptive Analyses

#### 3.1.1. Participant Information

The final sample selected for data analysis comprised 376 LGBTQ+ people. The sociodemographic characteristics are described in Table 2.

Sixty-one participants (16.2%) additionally declared being trans. Furthermore, physical self-reported characteristics of the participants are described in Table 3.

#### 3.1.2. Study Variables

Descriptive statistics about key study variables are described in Table 4.

Frequencies of specific experiences of vicarious trauma are described in Table 5, expressed in raw number of participants reporting it and percentage of the sample.

### 3.2. Correlational Analyses

Table 6 shows the correlations between the main study variables at the bivariate level.

All main variables were positively and significantly correlated with each other, ranging from small to large correlations. As could be expected, a particularly large correlation was found between shame and low self-esteem. Results were consistent with H_1_ through H_5_ at the bivariate level.

### 3.3. Mediation Analyses

The mediation model showed excellent fit, explaining 42.6% of the variance in the EDRC score (R^2^: 0.426, F = 31, *p* < 0.0000).

#### 3.3.1. Main Variables in the Regression Models

In accordance with H_1_ and H_2_, vicarious trauma showed a positive significant association with EDRC (B = 0.599, 95% CI [0.278, 0.921]), emotion dysregulation (B = 0.168, 95% CI [0.063, 0.273]), low self-esteem (B = 0.126, 95% CI [0.004, 0.249]) and shame (B = 0.328, 95% CI [0.159, 0.496]). In contrast with H_3_, the relationship between EDRC and shame was not found to be significant (95% CI [−0.128, 0.501]). In accordance with H_4_ and H_5_, positive significant associations were found between EDRC and emotion dysregulation (B = 0.476, 95% CI [0.082, 0.869]) as well as low self-esteem (B = 1.227, 95% CI [0.853, 1.601]).

#### 3.3.2. Covariates in the Regression Models

Among sociodemographic covariates, the results were mixed. The effect of BMI was nonsignificant on shame, low self-esteem, or emotion dysregulation, but a positive association was found for EDRC (B = 1.680, 95% CI [1.185, 2.174]). The effect of age was nonsignificant on EDRC, but a negative association was found for emotion dysregulation (B = −0.165, 95% CI [−1.683, −0.767]), low self-esteem (B = −0.135, 95% CI [−0.227, −0.043]), and shame (B = −0.215, 95% CI [−0.335, −0.095]). The effect of socioeconomic status was found to be nonsignificant for EDRC and shame, but a negative association was found with low self-esteem (B = −0.984, 95% CI [−1.770, −0.197]), and emotion dysregulation (B = −0.880, 95% CI [−1.683, −0.076].

#### 3.3.3. Total, Direct, and Indirect Effects

The total effect of the mediation model was found to be significant (B = 0.897, SE = 0.191, 95% CI [0.519, 1.274], *p* < 0.0000).

Among covariates, BMI (B = 1.680, SE = 0.251, 95% CI [1.185, 2.174) was positively correlated with eating disorder risk, and age was negatively correlated with eating disorder risk (B = −0.402, SE = 0.165, 95% CI [−0.719, −0.084]). Socioeconomic status was nonsignificant (95% CI [−4.011, 1.368]).

In accordance with H_1_, a positive direct effect of vicarious trauma was found on eating disorder risk (B = 0.599, SE = 0.163, 95% CI [0.278, 0.921]).

In accordance with H_2_, H_4_, H_5_, and in partial accordance with H_6_, positive indirect effects between vicarious trauma and eating disorder risk were found through low self-esteem (B = 0.155, BootSE = 0.080, 95% CI [0.001, 0.320]) and emotion dysregulation (B = 0.080, BootSE = 0.041, 95% CI [0.010, 0.168]). In contrast with H_3_ and partial contrast with H_6_, the indirect effect through shame was not significant (95% CI [−0.039, 0.178]).

**Figure 2:**
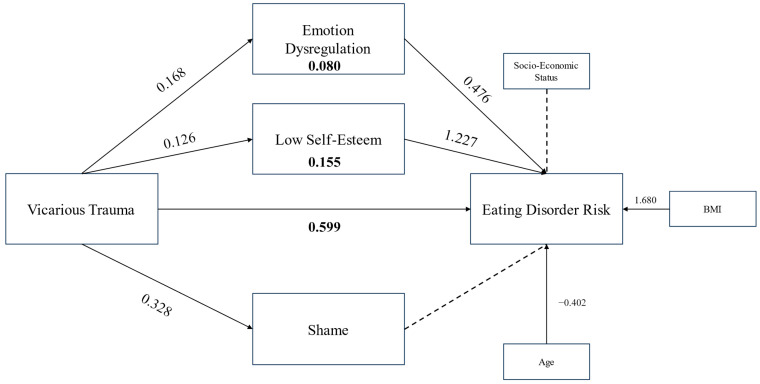
Measured parallel mediation model. Bold: direct/indirect effect. Dotted line: 95% CI included 0—relationship not statistically significant. BMI: Body mass index.

Figure 2 synthesizes the measured mediation model in a graphical representation.

## 4. Discussion

Most of the initial hypotheses of the study have been confirmed.

Vicarious trauma showed a significant direct effect on eating disorder risk, while two out of the three mediators highlighted partial mediation, coherently with the psychological mediation framework (Hatzenbuehler, 2009). Only the hypothesis regarding shame acting as a mediator was not verified.

Distress related to vicarious trauma was very common in our sample (mean score of 24.7 out of a maximum detected range of 30), depicting it as a salient experience that is near-universal in the study participants. The mediation analysis highlighted its negative effects on several intrapsychic characteristics as well as eating disorder risk. As suggested in previous studies (Herek et al., 1999; Noelle, 2002; Perry & Alvi, 2012), heterosexist experiences appear to have a negative effect on mental health even when experienced vicariously. In an international context in which LGBTQ+ people and LGBTQ+ rights are increasingly becoming the target of bias-motivated attacks and scapegoating by major political actors (Guillot & Coi, 2025; ILGA Europe, 2021), the experience of this type of minority stress is likely to trend further upwards and pose further risks to LGBTQ+ people’s health. This is particularly worrying for eating disorder risk in light of its mortality risk and health burden (Van Hoeken & Hoek, 2020).

Witnessing heterosexist experiences may induce vicarious trauma in people who share the salient LGBTQ+ identity, evoking feelings of fear, helplessness, shock, and anger as well as the sensation of being potentially threatened (Lannert, 2015; Sullaway, 2004). Consistently with minority stress theory, these feelings would also add to the feeling of needing to hide one’s identity (Meyer, 2003). These findings offer us additional interpretations for why it seems to increase emotion dysregulation in LGBTQ+ people. Emotion regulation capacity may be conceptualized as a potentially finite resource, which is depleted by having to frequently cope with negative affect linked to vicarious traumatization (i.e., “ego depletion”; Inzlicht et al., 2006). This, in turn, may lead to a heightening of eating disorder risk when depleted early through minority stressors. Vicarious trauma also seems to lower people’s self-esteem level. This may be linked to perceiving part of oneself to be stigmatized and rejected by society, as well as through being part of a group at risk of minoritization and exclusion. In addition to being a known risk factor for eating disorders (Colmsee et al., 2021; Mora et al., 2017), self-esteem may be further impacted in LGBTQ+ people by minority stress through lower social support and isolation (Closson & Comeau, 2021; Taylor et al., 2022).

In our model, shame was not a significant mediator. Despite showing no significant multicollinearity when conducting the assumptions check for linear regression, it is possible that the lack of statistical significance of shame in the model is due to a high level of shared variance with low self-esteem. The two constructs may share a significant amount of overlap regarding negative self-evaluation. Shame is a powerful emotion that is strongly linked to beliefs about evaluations coming from others (i.e., external shame) or oneself (i.e., internal shame; Goss & Allan, 2009). Shame proneness, as explored in this model, has been found to be an important aspect in eating disorder symptoms (Nechita et al., 2021) due to its role in causing and maintaining disordered eating behaviors. It can additionally prevent help-seeking behaviors, which can further endanger health outcomes. Self-esteem similarly refers to a global or specific evaluation of the self, which can be positive or negative, highlighting a stable self-concept (Rosenberg et al., 1995). It is possible that both may be important, as highlighted in previous studies, and that a study design able to assess each variable over time (i.e., longitudinal) would expose the role of shame in this process.

The significance of BMI, despite its limitations as a measure (e.g., lack of precision, inability to distinguish muscle from body fat), seems to have been confirmed—body dissatisfaction in particular has been reliably associated with weight status, of which BMI can be considered an imperfect proxy (Goldfield et al., 2010). As in previous literature (Rohde et al., 2017), age was also negatively related with eating disorder risk. The role of socioeconomic status was not significant, contrary to expectations from the literature which paints socioeconomic status as an important aspect in eating disorder risk, such as through the added stress of food insecurity and the way it shapes eating behaviors in people with low socioeconomic status (Rasmusson et al., 2019). It is possible that this is due to the homogeneity of the current sample, which primarily comprises people who perceive themselves to be of either sufficient or comfortable (79.8%) socioeconomic status.

Finally, it should be noted that grouping results from many different LGBTQ+ identities, as was carried out in this model, may obscure their specificities and differences. Each specific identity may have differing relationships with some of the included variables. For example, trans and gender-diverse people may have a particular relationship with thinness and muscularity due to the link between body image ideals and gender congruence, as bodily experiences strongly influence eating-related challenges (Santoniccolo et al., 2025).

### 4.1. Implications

The findings from this study further recontextualize heterosexism as a public health issue. As highlighted by international reports, Italy is lagging significantly behind in providing structural protections such as national laws against discrimination and hate crimes that involve sexual identity (Equaldex, 2025; ILGA Europe, 2025). Ideally, policies and structural protections should be put in place at the local, national, and international level that are aimed at preventing discrimination, violence, and stigma related to sexual orientation and gender identity, as they may protect the physical and mental health of LGBTQ+ people.

Previous studies on minority stress in the Italian context paint it as a prevalent, clinically relevant issue (Priola et al., 2014; Scandurra et al., 2017, 2020). Focusing on minority stress in clinical contexts has also been previously suggested to be a promising path (Frost & Meyer, 2023; Trombetta et al., 2024b, 2024c). Attempts have been made to develop treatments for disordered eating that are informed by minority stress theories with promising results: interventions may be aimed at strengthening resilience, conducting psychoeducation on minority stress, and promoting self-affirmation, while addressing body image concerns, dietary restraints, and affect regulation strategies (Brown et al., 2024). Working on relevant mediators of this relationship—in this case, emotion dysregulation and low self-esteem—may also mitigate the effects of vicarious trauma. For example, interventions focusing on emotion dysregulation may attempt to reduce rumination, improve metacognition, and work on acceptance of unwanted emotions (Leppanen et al., 2022). Additionally, interventions focusing on increasing self-esteem have been found to be effective in reducing eating disorder symptomatology (Kästner et al., 2019)—exploring how minority stress may have affected self-esteem and providing affirming care regarding sexual orientation and gender identity could be useful in this regard.

Programs promoting knowledge and information around LGBTQ+ identities may also prove useful in reducing the spread of negativity and promote contexts that protect individuals’ health through being respectful of diversities in sexual orientation and gender identity (Carpinelli et al., 2023).

### 4.2. Limitations and Future Directions

The study uses self-report measures, which have known limitations such as socially desirable responding (Allison & Heshka, 1993) or researcher acquiescence (Paulhus, 1991). Body mass index computation could also have suffered from distortions in self-reporting of height and weight (Krul et al., 2011).

The sample was also homogeneous in other sociodemographic characteristics, showing a young mean age, a relatively high level of education, and an overall sufficient socioeconomic status. As previously discussed (Trombetta et al., 2024a), more research on sexual and gender minorities with more diverse samples would be necessary to confirm these relationships. The sample’s young mean age may limit generalizability to older LGBTQ+ people (i.e., people in late adulthood and older). Low socioeconomic status in particular may be an additional axis of intersectional analysis (Crenshaw, 1991) and has previously been associated with higher chances of eating disorders in sexual and gender minorities (Burke et al., 2023). The role of multiple marginalizations related to eating behaviors in LGBTQ+ people may merit further attention.

Although our use of a non-probability sampling method (snowball sampling) is an efficient—although biased—way to reach statistical minority populations, it could also limit the generalizability of our findings. Future studies could improve upon this aspect by using probability sampling to gather participants.

The employed measure for eating disorder risk is a general one, rather than a specific one. Given the differences in etiopathology for eating disorders (Agras & Robinson, 2017), it may be interesting to explore these relationships in specific eating disorders (e.g., bulimia, anorexia) in future studies. Furthermore, employing a study design that uses a certified diagnosis as a dependent variable rather than general eating disorder risk may provide more precise estimations.

Additional studies on vicarious trauma would be needed to confirm these findings, as it remains a generally understudied facet of minority stress with limited existing evidence. Longitudinal studies in particular should be conducted to provide more reliable insights, as existing evidence is overwhelmingly cross-sectional (Santoniccolo & Rollè, 2024). The generalizability of these results to people outside these characteristics should be interpreted with caution and should be confirmed through further studies. Conducting studies on these relationships on specific identities (e.g., gay, lesbian, or bisexual people, trans men and women, etc.) may further deepen the understanding of this phenomenon. Furthermore, conducting future studies with larger sample sizes may help provide more certainty to the study’s findings. Additionally, while the causal order of the variables was theoretically defined and appears logically sound, the cross-sectional nature of the study makes it ill-fitted to provide certainty in the order of causal relationships. Finally, qualitative studies—exceedingly rare in this field (Santoniccolo & Rollè, 2024)—may prove useful in surveying in-depth the experiences of LGBTQ+ people regarding eating disorders and how they may be related to minority stress.

## 5. Conclusions

Distress related to LGBTQ+ vicarious trauma appears to heighten eating disorder risk and appears to achieve this through its impact on low self-esteem and emotion dysregulation. Reducing minority stress may prove to be important for reducing physical and mental health risks for LGBTQ+ people.

## Figures and Tables

**Table 1:** McDonald’s ω values for employed subscales.

Subscale	McDonald’s ω
Vicarious Trauma ^1^	0.787
Bulimia ^2^	0.893
Drive for Thinness ^2^	0.929
Body Dissatisfaction ^2^	0.885
Low Self-Esteem	0.911
Emotion Dysregulation	0.814
Shame	0.892

^1^: Translated in Italian. ^2^: Used in computing EDRC.

**Table 2:** Sociodemographic characteristics.

**Gender Identity**	**N**	**%**
Woman	190	50.5
Man	118	31.4
Non-binary	36	9.6
Gender non-conforming	15	4
Genderfluid	11	2.9
Other gender identity	6	1.6
**Sexual Orientation**	**N**	**%**
Bisexual	113	30.1
Gay	88	23.4
Lesbian	74	19.7
Pansexual	53	14.1
Asexual	24	6.4
Queer	13	3.5
Heterosexual *	7	1.9
Other sexual orientation	4	1.1
**Socioeconomic Status ^1^**	**N**	**%**
Insufficient	7	1.9
Precarious	56	14.9
Sufficient	187	49.7
Comfortable	113	30.1
More than well-off	8	2.1
**Education Status ^2^**	**N**	**%**
No formal education	0	0
Primary education	0	0
Lower secondary education/Middle school	3	0.8
Upper secondary education/Diploma	191	50.8
Bachelor’s degree/First-level university degree	104	27.7
Master’s degree/Second-level university degree	60	16
Doctorate/Specialization/Third-level university degree/Postgraduate studies	17	4.5
**Relationship status**	**N**	**%**
Single	174	46.3
Couple	113	30.1
Cohabiting couple	58	15.4
Married/Civil union	7	1.9
Polyamorous relationships/One or more non-monogamous relationships	18	4.8
Other relationship status	5	1.3

^1^: Five missing responses. Percentages of N = 371. ^2^: One missing response. Percentages of N = 375. *: People who reported diversities in their gender identity (e.g., being trans, non-binary, etc).

**Table 3:** Physical characteristics.

Variable	Range	Mean	SD
Age (in years) ^1^	18–57	25.82	6.29
Height (in centimetres)	152–194	169.31	8.61
Weight (in kilograms)	30–160	67.14	17.04
Body mass index	12–54	23.29	5.07

^1^: One missing response. SD: Standard deviation.

**Table 4:** Measured range, means, and standard deviations of the main variables.

Variable	Range	Mean	SD
Vicarious Trauma	6–30	24.7	5.13
Low Self-Esteem	0–24	10.1	6.32
Emotion Dysregulation	0–29	6.48	5.82
Shame	0–40	18.1	8.26
Eating Disorder Risk Composite	0–95	32.9	21.6

Values are expressed in non-standardized raw scores. SD: Standard deviation.

**Table 5:** Frequencies of experiences of vicarious trauma.

Vicarious Trauma Experience	N	%
Hearing about LGBT people you know being treated unfairly	283	75.3
Hearing about LGBT people you don’t know being treated unfairly	351	93.4
Hearing about hate crimes (e.g., vandalism, physical or sexual assault) that happened to LGBT people you don’t know	364	96.8
Hearing other people being called names such as “fag” or “dyke”	322	85.6
Hearing someone make jokes about LGBT people	359	95.5
Hearing politicians say negative things about LGBT people	371	98.7

**Table 6:** Two-tailed Pearson’s correlations.

Variable	Vicarious Trauma	Low Self-Esteem	Emotion Dysregulation	Shame	Eating Disorder Risk Composite
Vicarious Trauma	–				
Low Self-Esteem	0.109 *	–			
Emotion Dysregulation	0.159 **	0.501 ***	–		
Shame	0.213 ***	0.722 ***	0.523 ***	–	
Eating Disorder Risk Composite	0.192 ***	0.510 ***	0.374 ***	0.429 ***	–

*: *p* < 0.05; **: *p* < 0.01; ***: *p* < 0.001.

## Data Availability

The data that supports the findings of this study are available from the corresponding author Tommaso Trombetta upon reasonable request.

## Abbreviations

The following abbreviations are used in this manuscript:

LGBTQ+	Lesbian, Gay, Bisexual, Trans, Queer, and more
EDI-3	Eating Disorder Inventory—3
EDRC	Eating Disorder Risk Composite
PFQ-2	Personal Feelings Questionnaire—2
BMI	Body Mass Index
SD	Standard Deviation
